# Bidirectional Longitudinal Relations Between Parent–Grandparent Co-parenting Relationships and Chinese Children’s Effortful Control During Early Childhood

**DOI:** 10.3389/fpsyg.2020.00152

**Published:** 2020-02-25

**Authors:** Xiaowei Li, Siyu Zhou, Yuanfang Guo

**Affiliations:** Institute of Early Childhood Education, Faculty of Education, Beijing Normal University, Beijing, China

**Keywords:** effortful control, mother–grandparent co-parenting relationship, father–grandparent co-parenting relationship, maternal parenting self-efficacy, early childhood

## Abstract

Parent–grandparent co-parenting has become a common mode in Chinese families; however, its correlation with children’s development in the long run remains unclear. Herein, a 10-month follow-up survey was conducted among 253 preschool children and their parents from Chinese parent–grandparent co-parenting families. It aimed to examine the bidirectional longitudinal correlation of children’s effortful control with mother–grandparent and father–grandparent co-parenting relationships, as well as the dissimilarity of the two co-parenting relationships. In addition, the moderating role of maternal parenting self-efficacy in these relationships was also investigated. A cross-lagged model showed that the (1) mother–grandparent co-parenting relationship (T1) positively predicted the father–grandparent co-parenting relationship (T2), (2) dissimilarity of the mother–grandparent and father–grandparent co-parenting relationships (T1) negatively predicted children’s effortful control (T2), and (3) maternal parenting self-efficacy significantly moderated the predictive effect of children’s effortful control on a father–grandparent co-parenting relationship. However, a further simple slope analysis showed that after controlling the father–grandparent co-parenting relationship (T1), the children’s effortful control (T1) did not significantly predict the father–grandparent co-parenting relationship (T2) either in the high or low maternal parenting self-efficacy group. These results indicated that in Chinese parent–grandparent co-parenting families, the father-grandparent co-parenting relationship was influenced by the mother–grandparent co-parenting relationship, and similar mother–grandparent and father–grandparent co-parenting relationships were conducive to the development of the children’s effortful control.

## Introduction

*Effortful Control* is the ability to inhibit a dominant response and/or to activate a subdominant response, to plan, and to detect errors (Rothbart and Rueda, 2005). Children with a high level of effortful control are more likely to control their emotions and behaviors as well as to perform better in future social adjustment, school preparation, and academic achievement (Kochanska and Knaack, 2003; Eisenberg et al., 2005; Blair and Razza, 2010; Paulus et al., 2015).

As one of the important dimensions of temperament, effortful control is subject to great impacts from biological and genetic factors. Still, the role of the family environment should not be ignored (Eisenberg et al., 2005; Rothbart and Bates, 2006). Previous studies confirmed that both mother and father could help children learn emotional and behavioral control through appropriate parenting behaviors such as guidance, demonstration, and correction (Gartstein and Fagot, 2003; Eiden et al., 2004). However, according to the family system theory, all parenting behaviors in the family context are not isolated from each other (Bowen, 1966). Parents share the responsibility of raising children by building a co-parenting relationship (Feinberg, 2003). Such a situation may be even more complex in Chinese families with young children, where other roles in addition to the mother and father are involved in the co-parenting relationship. Owing to the dual pressure of work and life, young parents may not have enough time and energy to take care of their children. Meanwhile, most of the grandparents, having retired from workplaces, voluntarily participate in the raising of grandchildren because of the large amount of spare time, as well as the concern for younger generations. Thus, a typical co-parenting mode consisting of parents and grandparents has formed and become popular in China’s society these days (Li et al., 2016).

Theoretically, a co-parenting relationship can provide additional information about aspects of family functioning relevant to children’s developmental outcomes, which cannot capture the information on either parent’s individual behaviors (McHale et al., 1996). Empirically, Belsky et al. (1996) found that after controlling parental parenting behaviors, co-parenting could significantly predict a toddler’s behavioral inhibition. The study of Karreman et al. (2008) also revealed the unique predictive effect of co-parenting on children’s effortful control after controlling parental parenting. The results of these previous studies together showed that co-parenting contributed to the children’s effortful control over and above parenting, which indicated that co-parenting has a unique value in explaining a significant proportion of the variance of the children’s effortful control. Based on that, a wide range of literature has substantiated the close connection between the mother–father co-parenting relationship and children’s effortful control without taking either parent’s individual behaviors into account (Schoppe-Sullivan et al., 2009; Burney and Leerkes, 2010; Metz et al., 2016; Jahromi et al., 2017). Analogous to the mother–father co-parenting relationship, the parent–grandparent co-parenting relationship equally serves as a key component of the family system and may also be related to the children’s effortful control. Notably, most relevant studies on parent–grandparent co-parenting relationship in western countries focused on disadvantaged families, whose results hence cannot be directly extended in general to Chinese families (Barnett et al., 2011, 2012). Though some domestic scholars explored the current situation of the parent–grandparent co-parenting relationship and its influence on children’s development, the majority of studies are cross-sectional studies (Li et al., 2016; Xing et al., 2016). Only a few works paid attention to the longitudinal correlation between the parent–grandparent co-parenting relationship and children’s effortful control in Chinese families. Thus, this longitudinal correlation was explored herein targeting the general Chinese parent–grandparent co-parenting families.

### Co-parenting Relationship and Children’s Effortful Control

Existing empirical studies extended their support for the bidirectional correlations between mother–father co-parenting relationship and children’s effortful control. On one hand, the quality of mother–father co-parenting relationship was found to have an impact on the level of children’s effortful control. Co-parenting conflicts may increase negative emotional expression in the family environment, which would trigger young children’s high-level negative emotional arousal, which would occupy their psychological resources originally used for self-regulation. Meanwhile, such tense family environment would stimulate children’s self-protection mechanism such as escape, so as to hinder them from learning and modeling good emotional and behavioral adjustment strategies from parents. Instead, children growing up in such a family environment are forced to observe more arguments and conflicts between parents and unavoidably imitate to deal with problems in an unpleasant way (Halberstadt et al., 1999; Karreman et al., 2008; Merwin et al., 2017). On the other hand, some scholars concluded that children’s effortful control may also influence the quality of mother–father co-parenting relationship. The care for children with a higher level of effortful control was found to be easier, encouraging parents to form a positive co-parenting relationship. On the contrary, children with a lower level of effortful control may put more parenting stress on both parents, eventually leading to a negative mother–father co-parenting relationship (Lindsey and Colwell, 2005; Schoppe-Sullivan et al., 2007; Burney, 2010; Burney and Leerkes, 2010).

The same as the mother–father co-parenting relationship, the parent–grandparent co-parenting relationship is another kind of co-parenting relationship in the family system among family members who participating in child raising. A few recent cross-sectional studies preliminarily indicated the possible correlations between the parent–grandparent co-parenting relationship and children’s developmental outcomes (Li et al., 2016; Li and Wei, 2018). Based on the similar mechanism of the bidirectional correlations between the mother–father co-parenting relationship and children’s effortful control mentioned above, in the current study, the parent–grandparent co-parenting relationship was further assumed to have bidirectional longitudinal correlations with children’s effortful control in China’s parent–grandparent co-parenting families.

### Mother–Grandparent Co-parenting Relationship and Father–Grandparent Co-parenting Relationship

Since parent–grandparent co-parenting has become a common mode in Chinese families, some recent studies have paid attention to this specific topic area (Li et al., 2016; Li and Wei, 2018). However, most of these studies focused on the mother–grandparent co-parenting relationships. The father–grandparent co-parenting relationship was traditionally ignored (Li and Wei, 2018). However, as Chinese fathers got more and more involved in family education (Li and Wei, 2017), establishing co-parenting relationships with grandparents is no longer a problem that only mothers have to face these years. Li et al.’s (2016) empirical study showed that fathers may build up different co-parenting relationships with grandparents in comparison to mothers, which suggested the necessity to investigate the father–grandparent co-parenting relationship separately. Furthermore, the family system theory suggests that all participants in the family system are interdependent and interacted with each other (Bowen, 1966; Minuchin, 1985), which indicates the interaction between the mother–grandparent and father–grandparent co-parenting relationships. Such interaction, however, has not been empirically substantiated until now.

Therefore, based on the scores of the parent–grandparent co-parenting relationship reported by both parents, this study not only examined the different correlations of the children’s effortful control with the mother–grandparent and father–grandparent co-parenting relationships but also explored the interactive predictive effect between the mother–grandparent and father–grandparent co-parenting relationships.

### Dissimilarity of Mother–Grandparent and Father-Grandparent Co-parenting Relationships and Children’s Effortful Control

The scores of the parent–grandparent co-parenting relationship reported by both parents allow researchers to calculate the dissimilarity of the mother–grandparent and father–grandparent co-parenting relationships, which may be another predictor for children’s effortful control (Zhang et al., 2009). A few studies were carried out on links between marital satisfaction and mother–father co-parenting relationship (Schoppe-Sullivan et al., 2007; Pedro et al., 2012; Liu et al., 2016). For instance, a recent study of Liu et al. showed that a high similarity of marital satisfactions between two parents contributed to alleviation of co-parenting conflicts (Liu et al., 2016). A high marital satisfaction reported by both parents indicated the high-quality intimacy of the couples and their ability to solve conflicts and problems in co-parenting relationship through effective communication (Schoppe-Sullivan et al., 2004; Bonds and Gondoli, 2007). By contrast, a low marital satisfaction reported by both parents presented that the couples were in the cold war, during which all the parenting interaction, including co-parenting conflicts, got reduced due to the lack of communication (Liu et al., 2016). Such correlation may also exist in the mother–grandparent and father–grandparent co-parenting relationships. To be more specific, the phenomenon that both parents reported a high-quality co-parenting relationship with grandparents may indicate fewer co-parenting conflicts among all the family members. On the contrary, the fact that both parents reported a low-quality co-parenting relationship with the grandparents may reflect the couples’ equal dissatisfaction with the grandparents’ participation in the child-raising process. Hence, it suggested a necessity to understand each other and establish a strong, positive co-parenting alliance that is conducive to the development of the children’s effortful control.

Moreover, compared with the mother, the father is more susceptible to the children’s difficult temperament and, therefore, reports a lower score for the co-parenting relationship (Sirignano and Lachman, 1985; McBride and Rane, 2002; Burney and Leerkes, 2010; Solmeyer and Feinberg, 2011). This tends to enlarge the gap between the mother–grandparent and father–grandparent co-parenting relationships. Hence, it was assumed here that, on one hand, dissimilarity of the mother–grandparent and father–grandparent co-parenting relationships could be used to predict the level of the children’s effortful control; one the other hand, the level of the children’s effortful control provided a reference in determining dissimilarity of the mother–grandparent and father–grandparent co-parenting relationships.

### The Moderating Role of Maternal Parenting Self-Efficacy

As the most principal caregiver of children, the mother plays a vital role in the family system. As proved by a number of studies, fathers of hyperactive infants were more likely to report a negative co-parenting relationship; such tendency may be reduced by their effective communication with spouses (Burney and Leerkes, 2010; Gordon and Feldman, 2010; Laxman et al., 2013). It reflected that the correlation of children’s temperament with father-grandparent co-parenting relationship may be moderated by some mother-related factors. Furthermore, some papers illustrated that a mother with high parenting self-efficacy could take positive parenting styles to promote the children’s development of effortful control (Kim, 2015) and establish a good co-parenting relationship with her husband (Kwan et al., 2015; Arguello, 2017). Such positive mother–father co-parenting relationship may “spill over” into the parent–grandparent co-parenting relationship according to the family system theory. In other words, when faced with children having a low level of effortful control, a mother with high parenting self-efficacy may promote the effective communication between the father and grandparents, the coordination of co-parenting relationships among all family members, and the unity of all caregivers to face the parenting challenges. This will alleviate the negative influence of low-level children’s effortful control on father–grandparent co-parenting relationship. As a result, it was assumed in this paper that maternal parenting self-efficacy played a role in moderating the predictive impact of children’s effortful control on father–grandparent co-parenting relationship.

### The Present Study

Based on the above assumptions, the bidirectional longitudinal correlations of children’s effortful control with mother–grandparent and father–grandparent co-parenting relationships, as well as the dissimilarity of the two co-parenting relationships were examined using the cross-lagged model. Furthermore, the moderating role of maternal parenting self-efficacy in the predictive effect of children’s effortful control on father–grandparent co-parenting relationship was investigated by means of the regression linear equation.

## Materials and Methods

### Participants

Participants were recruited from a preschool in Guangzhou, China. To screen parent–grandparent co-parenting families, we highlighted the definition of parent–grandparent co-parenting in the instructions of a questionnaire and asked parents to complete the questionnaire only when the grandparents are participating in the raising of the grandchildren. Finally, the parents of 324 preschool children from parent–grandparent co-parenting families completed questionnaires in the first survey (T1). Ten months later (T2), the parents of 253 children continually participated in the follow-up survey (78.09% of the original sample). Attrition was mainly due to the high dropout rate of Chinese children in their final year of preschool. Attrition analysis showed no significant differences in demographic and all study-relevant variables between the longitudinal sample and attrition sample.

The final sample of 253 children whose parents participated in both T1 and T2 surveys was 54.90% male and had a mean age of 4.27 years at T1 (*SD* = 0.91, range: 3–6 years). Of the children, 73.7% were the only child in their family, and 79.9% of the children lived in three-generation families with both parents and grandparents. In 132 (52.2%) parent–grandparent co-parenting families, the main grandparent who participated in the child rearing was the parental grandmother. Eighty-four (33.3%) were maternal grandmothers. The number of parental and maternal grandfather was 26 (10.3%) and 11 (4.3%), respectively. The average age of the mothers was 33.95 years old (*SD* = 2.63, range: 26–42 years), and 81.8% had a college degree or above. The average age of the fathers was 36.27 years old (*SD* = 2.87, range: 30–48 years), and 74.7% had a college degree or above. The average age of the grandparents involved in co-parenting was 60.91 years old (*SD* = 5.04, range: 42–80 years), and 94% had a high school degree or less. Furthermore, 53.5% of the participating families had an annual income of RMB 100,000 (about $14,400 USD) or above that belonging to the middle- or high-income families according to the National Statistical Bureau of the People’s Republic of China (2018).

### Procedures

Ethics approval for the current study was obtained from the Ethics Committee of the School of Psychology, Beijing Normal University. Questionnaires were distributed in a preschool in Guangzhou, which is the third largest city in China. We randomly selected the sample preschool from the list of Guangzhou preschools by a clustered random sampling method. After obtaining the consent of the preschool principal, the informed consent forms and questionnaires were issued to the parents by experienced researchers and class teachers together when parents picked up their children, and the notes to complete the questionnaires were explained to the parents at the same time. The parents are required to seal the completed questionnaires in an envelope and return them to the class teachers within 1 week, after which, the researchers gathered all the returned questionnaires. The first survey was conducted at the beginning of the new semester in September (T1), and the follow-up survey was conducted 10 months later in June of the next year (T2). SPSS 21.0 and Mplus 7.4 were used for the subsequent statistical analysis.

### Measures

#### Parent–Grandparent Co-parenting Relationship

At T1 and T2, both parents were asked to completed the revised Chinese version of the Parent–Grandparent Co-Parenting Relationships Scale (Li and Wei, 2018), which was adapted from the Co-Parenting Relationships Scale (CRS) originally developed to assess mother–father co-parenting relationships (Feinberg et al., 2012), to rate their own co-parenting relationship with grandparents. The scale includes 35 items in seven dimensions (co-parenting agreement, co-parenting closeness, exposure of child to conflict, co-parenting support, endorsement of partner’s parenting, co-parenting undermining, and division of labor), and each item was rated on a seven-point scale, ranging from 1 (completely inconsistent) to 7 (completely consistent). At T1, the *Cronbach’s* α of mother-reported scores of the seven dimensions ranged from 0.63 to 0.85. The *Cronbach’s* α of father-reported scores of the seven dimensions ranged from 0.67 to 0.85. At T2, the *Cronbach’s* α of mother-reported scores of the seven dimensions ranged from 0.65 to 0.88. The *Cronbach’s* α of mother-reported scores of the seven dimensions ranged from 0.62 to 0.87.

The average of the total score of each dimension was calculated, with higher scores reflecting a more positive parent–grandparent co-parenting relationship. Dissimilarity of mother–grandparent and father–grandparent co-parenting relationships was represented by the absolute value of the difference between the scores of father–grandparent co-parenting relationship and mother-grandparent co–parenting relationship. Higher scores indicated a greater dissimilarity of mother–grandparent and father–grandparent co-parenting relationships (Zhang et al., 2009).

#### Children’s Effortful Control

At T1 and T2, the mothers completed the 36-item Very Short Form of Children’s Behavior Questionnaire, designed to assess three broad temperament factors (Surgency, Negative Affect, and Effortful Control) in children aged 3–8 using a seven-point rating scale (1 = completely inconsistent; 7 = completely consistent) (Rothbart et al., 2001). The Chinese version of the questionnaire has been revised in Chinese culture and was proven to have good reliability and validity in the measurement of Chinese children’s temperament (Liang, 2011). For the purposes of this study, we only calculated the average of the 12-item scores of the Effortful Control scale, with higher scores indicating a higher level of children’s effortful control (*Cronbach’s* α = 0.72 at T1 and 0.83 at T2).

#### Maternal Parenting Self-Efficacy

At T1, the mothers completed the 31-item Parenting Self-efficacy Scale to rate their own parenting self-efficacy on a six-point scale, ranging from 1 (very unconfident) to 6 (very confident) (Holloway et al., 2005). The Chinese version scale has been proven reliable and valid in the previous studies to measure the parenting self-efficacy of Chinese preschool children’s mothers (Li and Wei, 2018). In the current study, the *Cronbach’s* α of the two dimensions of the scales (General Parenting Self-Efficacy and Specific Parenting Self-Efficacy) were 0.84 and 0.92. The average score of the 31-item scores was calculated, with higher scores indicating higher maternal parenting self-efficacy.

### Data Analysis

First of all, missing data were handled using the full information maximum likelihood estimation method, after which descriptive statistics and bivariate correlations were carried out in SPSS 21.0. To examine the bidirectional longitudinal correlations of children’s effortful control with mother–grandparent and father–grandparent co-parenting relationships, as well as the dissimilarity of the two co-parenting relationships, a set of autoregressive cross-lagged analyses were conducted within a structural equation modeling (SEM) framework using Mplus 7.4 (Mutheén and Mutheén, 1998–2012). The model is considered to be good when the comparative fit index (*CFI*) and Tucker–Lewis index (*TLI*) ≥ 0.90 (≥0.95 is ideal), root mean square error of approximation (*RMSEA*) ≤ 0.08 (≤0.06 is ideal), and standardized root mean square residual (*SRMR*) ≤ 0.08 (Hu and Bentler, 1999; Kline, 2011). Simple slop analysis was conducted then to further explore the moderating role of maternal parenting self-efficacy.

## Results

### Descriptive Statistics

Table 1 presents the means, standard deviations, and correlations for all study variables at T1 and T2. The results showed that (1) the scores of the children’s effortful control, mother–grandparent co-parenting relationship, and father–grandparent co-parenting relationship at T1 were all significantly positively correlated with their own scores at T2 (*rs* = 0.44 to 0.68); (2) the mother–grandparent co-parenting relationship was positively correlated with the father–grandparent co-parenting relationship both at T1 and T2 (*rs* = 0.43 and 0.51, respectively); (3) both the mother–grandparent co-parenting relationship and father–grandparent co-parenting relationship at T1 were significantly positively correlated with the children’s effortful control at T2 (*rs* = 0.13 and 0.13, respectively). Conversely, the children’s effortful control at T1 was significantly positively correlated with both the mother–grandparent co-parenting relationship and father–grandparent co-parenting relationship at T2 (*rs* = 0.13 and 0.13, respectively); (4) the dissimilarity of the mother–grandparent and father–grandparent co-parenting relationships at T1 was negatively correlated with the children’s effortful control at T2 (*r* = −0.17), while the correlation between the children’s effortful control at T1 and the dissimilarity of the mother–grandparent and father–grandparent co-parenting relationships at T2 was not significant (*r* = −0.04); and (5) the maternal parenting self-efficacy at T1 significantly positively correlated with the children’s effortful control, mother–grandparent co-parenting relationship, and father–grandparent co-parenting relationship at T1 and T2 (*rs* = 0.17 to 0.30).

**TABLE 1:** Means, standard deviations, and correlations among demographic and study variables at T1 and T2 (*N* = 253).

	**1**	**2**	**3**	**4**	**5**	**6**	**7**	**8**	**9**	**10**	**11**
1. Children age (T1)	–										
2. Children gender	–0.08	–									
3. Mother–grandparent co-parenting relationship (T1)	–0.02	–0.06	–								
4. Father–grandparent co-parenting relationship (T1)	0.13*	–0.02	0.43***	–							
5. Children’s effortful control (T1)	0.13*	0.19**	0.12	0.14*	–						
6. Dissimilarity of mother–grandparent and father–grandparent co-parenting relationships (T1)	–0.01	–0.04	−0.21**	0.12	–0.04	–					
7. Maternal parenting self-efficacy (T1)	0.11	–0.04	0.17**	0.18**	0.21***	0.02	–				
8. Mother–grandparent co-parenting relationship (T2)	0.04	–0.04	0.68***	0.35***	0.13*	−0.15*	0.13*	–			
9. Father–grandparent co-parenting relationship (T2)	–0.03	–0.08	0.42***	0.68***	0.13*	0.03	0.20**	0.51***	–		
10. Children’s effortful control (T2)	0.12	0.10	0.13*	0.13*	0.44***	−0.17**	0.30***	0.20**	0.29***	–	
11. Dissimilarity of mother–grandparent and father–grandparent co-parenting relationships (T2)	–0.05	0.08	–0.06	0.15*	–0.04	0.31***	0.12	−0.15*	0.15*	0.06	–
*M*	4.27	1.45	11.26	11.50	4.69	2.28	4.82	11.20	11.50	4.97	2.18
*SD*	0.91	0.50	2.74	2.64	0.40	17.5	0.51	3.01	2.64	0.53	1.79

***p < 0.001. *M*, mean; *SD*, standard deviations; T1, time 1; T2, time 2.

Concerning all of the demographic variables, we only found that children’s gender and age were significantly correlated with children’s effortful control at T1 (*rs* = 0.13 to 0.19), so children’s gender and age were used as control variables in the following analysis.

### Model Testing

**FIGURE 1:**
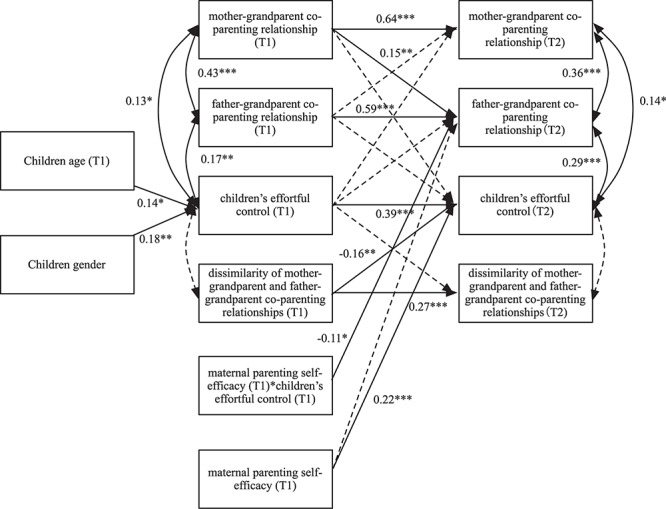
Cross- lagged structural equation model on the bidirectional relations among mother–grandparent co-parenting relationship, father–grandparent co-parenting relationship, dissimilarity of mother–grandparent and father–grandparent co-parenting relationships and children’s effortful control from T1 to T2 moderated by maternal parenting self-efficacy at T1. Solid lines indicate significant standard paths; dashed lines indicate non-significant paths. Only significant path coefficients are shown. *^∗^p* < 0.05, *^∗∗^p* < 0.01, *^∗∗∗^p* < 0.001. T1, time 1; T2, time 2.

After including all the autoregressive paths and concurrent correlations, multiple cross-lagged paths from each variable at T1 to all the other variables at T2 were added in the model (see Figure 1). In addition, maternal parenting self-efficacy at T1 and its interaction item with children’s effortful control was also included in the model in order to test the moderating role of maternal parenting self-efficacy on the predictive effect of children’s effortful control (T1) on father–grandparent co-parenting relationship (T2). After controlling the children’s gender and age, the cross-lagged model proved a good fit; χ*^2^*(23) = 23.91, *CFI* = 0.996, *TLI* = 0.993, *RMSEA* = 0.02, *SRMR* = 0.03, Δχ*^2^*(1) = 21.87, *p* < 0.01.

The path analyses showed that after controlling the children’s gender, age, and stability of variables (themselves) across time, (1) the mother–grandparent co-parenting relationship at T1 significantly positively predicted the father–grandparent co-parenting relationship at T2 (β = 0.15, *p* < 0.01), while no significant predictive effect of the father–grandparent co-parenting relationship at T1 on the mother–grandparent co-parenting relationship at T2 was found; (2) neither the mother–grandparents co-parenting relationship nor father–grandparents co-parenting relationship at T1 was found to significantly predict the children’s effortful control at T2 in the current study. Dissimilarity of the mother–grandparent and father–grandparent co-parenting relationships at T1 significantly negatively predicted the children’s effortful control at T2 (β = −0.16, *p* < 0.001), while we did not find that the children’s effortful control at T1 predicted the mother–grandparent co-parenting relationship, father–grandparent co-parenting relationship, or dissimilarity of the mother–grandparent and father–grandparent co-parenting relationships at T2; (3) maternal parenting self-efficacy at T1 significantly positively predicted the children’s effortful control at T2 (β = 0.22, *p* < 0.001). In addition, its interaction item with the children’s effortful control at T1 significantly negatively predicted the father–grandparent co-parenting relationship at T2 (β = −0.11, *p* < 0.05), which suggested that maternal parenting self-efficacy at T1 moderated the predictive effect of children’s effortful control at T1 on the father–grandparent co-parenting relationship at T2.

### Moderating Role of Maternal Parenting Self-Efficacy

To further explore the moderating role of maternal parenting self-efficacy in the predictive effect of children’s effortful control on father–grandparent co-parenting relationship, we divided the samples into high maternal parenting self-efficacy group and low maternal parenting self-efficacy group by adding/subtracting one standard deviation to/from the mean score of the maternal parenting self-efficacy. Then, hierarchical regression analyses were conducted on each group. The children’s gender, age, and father–grandparent co-parenting relationship at T1 were entered in the first step as the control variables, followed by the children’s effortful control (T1) in the second step. As shown in Table 2, the children’s effortful control (T1) was positively associated with the father–grandparent co-parenting relationship (T2) in the low maternal parenting self-efficacy group but negatively associated to the father–grandparent co-parenting relationship (T2) in the high maternal parenting self-efficacy group. Specifically, for each point reduced on the effortful control for children in the low maternal parenting self-efficacy group at T1, their father–grandparent co-parenting relationship at T2 reduced by 0.09 points, but for children in the high maternal parenting self-efficacy group, their father–grandparent co-parenting relationship at T2 increased by 0.12. Nevertheless, since the predictive effect of the children’s effortful control (T1) on the father–grandparent co-parenting relationship (T2) was statistically insignificant either in the high or low maternal parenting self-efficacy group, it was unconvincing for the current study to assert that maternal parenting self-efficacy could significantly alleviate the negative impact of a low level of children’s effortful control on the father–grandparent co-parenting relationship.

**TABLE 2:** Regressions predicting father–grandparent co-parenting relationship at T2 from children’s effortful control at T1 in the low and high maternal parenting self-efficacy groups.

		**Father–grandparent co-parenting**			**Father–grandparent co-parenting**		
		**relationship (T2) of low maternal**			**relationship (T2) of high maternal**		
		**parenting self-efficacy**			**parenting self-efficacy**		
		**group (T2, *n* = 47)**			**group (T2, *n* = 44)**		
			
**Step**	**Independent variable**	***B***	**SE**	**β**	***B***	**SE**	**β**
1	Children gender	0.61	0.56	0.12	−0.43	0.69	−0.08
	Children age	0.15	0.29	0.06	−0.08	0.39	−0.02
	Father–grandparent co-parenting relationship (T1)	0.78	0.11	0.76***	0.73	0.14	0.64
		*R*^2^ = 0.55, *F* = 16.98***			*R*^2^ = 0.44, *F* = 9.80***		
2	Children’s effortful control (T1)	0.62	0.90	0.09	−0.78	0.84	−0.12
		Δ*R*^2^ = 0.01, Δ*F* = 0.47			Δ*R*^22^ = 0.01, Δ*F* = 0.87		

***p < 0.001. T1, time 1; T2, time 2.

## Discussion

Herein, the bidirectional longitudinal relations of children’s effortful control with mother–grandparent and father–grandparent co-parenting relationships, as well as the dissimilarity of the two co-parenting relationships were explored. In addition, the moderating role of maternal parenting self-efficacy in the predictive impact of children’s effortful control on father–grandparent co-parenting relationship was investigated. First, it was found that the mother–grandparent co-parenting relationship played a significantly positive role in predicting the father–grandparent co-parenting relationship; but it was free from the impact of the latter relationship. That is to say, the father had more possibilities to keep a positive co-parenting relationship with the grandparents under the premise of good mother–grandparent co-parenting relationship. Nevertheless, it was hard for the father–grandparent co-parenting relationship to influence the co-parenting relationship of the mother with the grandparents. Such result was consistent with the reality of the Chinese parent–grandparent co-parenting families. As the most principal caregiver of children, the mother actually interacts with grandparents more frequently than the father in the child-raising process. Different parenting concepts make intergenerational co-parenting conflicts inevitable, especially between mother-in-law (grandmother) and daughter-in-law (mother) in context of the Chinese traditional culture (Shi, 2012). As a result, the father tends to receive the complaints from both his wife and his mother. The mother may complain to her husband about the grandparents’ unsatisfactory parenting behaviors, which hence negatively affect co-parenting relationship of the father with the grandparents. Meanwhile, the grandparents’ dissatisfaction with the mother will influence their evaluation for the father in the aspect of children parenting, thereby leading to a negative impact on the father–grandparent co-parenting relationship.

No significant predictive impact of the mother–grandparent or father–grandparent co-parenting relationships at T1 on the children’s effortful control at T2 was found in the current study. Instead, the investigated children showed a lower level of effortful control at T2 under a greater dissimilarity of the mother–grandparent and father–grandparent co-parenting relationships at T1. As demonstrated by literature, the externalizing co-parenting conflicts may trigger a high-level negative emotional arousal of young children, thus consuming their psychological energy originally used for effortful control and increasing the frequency of children’s emotional and behavioral regulation problems (Karreman et al., 2008). However, subject to the Chinese traditional culture that highly emphasizes filial piety and respect for the elderly, parents in Chinese parent–grandparent co-parenting families tend to avoid direct conflicts with grandparents for their elder status. Hence, the externalizing co-parenting conflicts between grandparents and parents may not directly manifest in the family environment as frequently as the mother–father co-parenting conflicts. However, when the mother and father report significantly different co-parenting relationships with the grandparents, it becomes possible for the invisible co-parenting conflicts between the parents and grandparents to transform into the externalizing conflicts between the parents. This will increase the negative emotional expression in the family environment, eventually not only occupying the psychological resources children originally used for self-regulation and stimulating the children’s self-protection mechanism such as escape (Halberstadt et al., 1999; Merwin et al., 2017) but also hindering them from learning good emotional and behavioral adjustment strategies from their parents. As a result, it may be difficult for children to develop a high level of effortful control (Burrowes and Halberstadt, 1987; Tan and Smith, 2018). In addition to the externalizing interparental conflicts and negative emotional expression in the family environment, dissimilarity of the parent–grandparent co-parenting relationships between parents might also link to other interparental factors, which can influence the development of the children’s effortful control. For instance, the parents’ different attitudes toward the grandparents’ parenting behaviors may trigger their dissatisfaction to each other, which would lead to a low level of marital adjustment. In this case, both parents may experience more emotional depression, feel increasing parenting stress, and adopt more negative parenting behaviors, which would threaten the children’s development of effortful control (Gartstein and Fagot, 2003; Ponnet et al., 2013; Lin et al., 2017; Taraban et al., 2017). Although the reliability and validity of the above possible explanations still wait to be substantiated, they all indicate a potential research direction for future studies that intergenerational co-parenting relationships may influence children’s developmental outcomes through some kinds of interparental relationships, which is also consistent with the family system theory that all relationships in the family system are interdependent and interacted with each other (Bowen, 1966).

Nevertheless, in the current study, children’s effortful control at T1 showed no significant predictive effect on mother–grandparent and father–grandparent co-parenting relationship as well as on the dissimilarity of the two co-parenting relationships at T2, which was inconsistent with the assumption mentioned before and the results of previous studies (Lindsey and Colwell, 2005; Schoppe-Sullivan et al., 2007; Burney, 2010; Burney and Leerkes, 2010). A possible explanation is given below. Children’s effortful control, as an important dimension of temperament, is under the impact of genetic factors and has strong stability across time. In addition, co-parenting relationship is proved to be a stable parenting variable across time as well (Laxman et al., 2013). The 10-month following-up investigation in the current study may be not sufficient to reveal the direct bidirectional relations between them.

Moreover, although the current study preliminarily revealed the possible alleviating effect of maternal parenting self-efficacy on the negative impact of low level of children’s effortful control on father–grandparent co-parenting relationship, such finding appears to be unconvincing since the predictive effect of children’s effortful control (T1) on father–grandparent co-parenting relationship (T2) was not statistically significant either in the high or low maternal parenting self-efficacy group. Such result, on one hand, may be attributed to the relatively weak predictive effect of children’s effortful control (T1) on father–grandparent co-parenting relationship (T2) after controlling father–grandparent co-parenting relationship (T1) because of the time stability of the two variables. On the other hand, according to the results of *post hoc* power analyses, the Effect Sizes (i.e. represented by *f*^2^ in multiple linear regression) for this specific predictive effect in both groups (0.02 and 0.01, respectively), were also small (Cohen, 1969), which indicated that the true predictive effect could exist, but our study was statistically unpowered to detect it given the relatively small sample size of each group (44 and 47, respectively). No matter which reason was, a longer follow-up study implemented in a larger sample is expected in the future to further substantiate the possible alleviating effect of maternal parenting self-efficacy on the negative impact of a low level of children’s effortful control on father–grandparent co-parenting relationship, which has preliminarily emerged in the current study.

This study has some limitations. First, a 10-month follow-up study may be too short to reveal the significant changes in parent–grandparent co-parenting relationship and children’s effortful control across time because both children’s effortful control and co-parenting relationship were proven to have strong time stability (Laxman et al., 2013). Longer time may be needed to observe their significant change. Second, children’s effortful control in this study was only reported by the mother, which was susceptible to bias. However, the mother-reported children’s effortful control could help to learn the overall general performance of children at home. After all, if knowing that they were being observed, children may deliberately make a better performance than their normal level, which would also induce bias. Therefore, it is necessary to adopt both tests and mother reports for measuring children’s effortful control. Third, the sample size of the current study was relatively small, which could be attributed to the follow-up study design and the screen for parent–grandparent co-parenting families. Such small sample size might reduce the statistical power of the current study. Therefore, caution should be warranted when interpreting and generalizing the study results. Especially the testing of the moderating role of maternal parenting self-efficacy, the number of children in high and low maternal parenting self-efficacy groups was only 44 and 47, respectively, which indicated a small statistical power and would consequently lead us to miss the statistical significant results for the possible predictive effect of children’s effortful control (T1) on father–grandparent co-parenting relationship (T2) in both groups. Finally, the current study did not include mother–father co-parenting relationship as a controlled variable in the cross-lagged longitudinal model, although it was also found to be closely related to children’s effortful control (Schoppe-Sullivan et al., 2009; Burney and Leerkes, 2010; Jahromi et al., 2017). Because simply adding mother–father co-parenting relationship in the model as a covariate ignoring the possible interaction between mother–father co-parenting relationship and parent–grandparent relationship may lead to the multicollinearity problem among independent variables (Farrar and Glauber, 1967), which would impede us to detect the unique effect of parent–grandparent co-parenting on children’s effortful control. However, it is still recommended for future studies to take mother–father co-parenting relationship in account when examining the relationship between parent–grandparent co-parenting and children’s developmental outcomes because according to the results of the current study, the dissimilarity of parent–grandparent co-parenting relationships between parents may trigger the parents’ externalizing conflicts including mother–father co-parenting conflicts, which indicated the potential interaction between mother–father co-parenting relationship and parent–grandparent relationship and their possible combined effect on children’s developmental outcomes.

## Conclusion and Implications

In conclusion, the current study enriched the existing study results on the topic area of parent–grandparent co-parenting relationship by providing additional data of father–grandparent co-parenting relationship in Chinese parent–grandparent co-parenting families. The study results can be widely implicated in the Chinese family education practice. According to the influence of mothers’ co-parenting relationship with grandparent on fathers’, parents, especially mothers, in parent–grandparent co-parenting, families should not only focus on their own co-parenting relationship with grandparents but also pay attention to their spouse’s co-parenting relationship with grandparents. In this case, parents would communicate more about their attitudes toward grandparents’ parenting behaviors so as to keep their co-parenting relationships with grandparents similar to each other, which would facilitate children’s development of effortful control. In addition, although the current study focused on Chinese parent–grandparent co-parenting families, we introduced more kinds of co-parenting relationships beyond mother–father co-parenting relationship in the topic area of co-parenting, which may inspire future studies across different cultures to think beyond mother–father co-parenting relationship and pay attention to different kinds of co-parenting relationships among multiple child-rearing participants (e.g. grandparents, teachers, and babysitters), which may interact with each other and have combined effects on children’s developmental outcomes.

## Data Availability Statement

The datasets generated for this study are available on request to the corresponding author.

## Ethics Statement

This study was carried out in accordance with the recommendations of the Ethics Committee of the School of Psychology, Beijing Normal University. The protocol was approved by the Ethics Committee of the School of Psychology, Beijing Normal University. Written informed consent was obtained from all participants. For all the children who participated in the study, written informed parental consent was obtained from their parents/legal guardians in accordance with the Declaration of Helsinki.

## Author Contributions

XL designed and executed the study, and assisted with the data analyses. SZ analyzed the data and wrote the manuscript. YG wrote part of the introduction and assisted with the data analyses.

## Conflict of Interest

The authors declare that the research was conducted in the absence of any commercial or financial relationships that could be construed as a potential conflict of interest. The reviewer XL declared a shared affiliation, with no other collaboration, with the authors to the handling Editor at the time of review.
